# Outlier detection in cardiac diffusion tensor imaging: Shot rejection or robust fitting?

**DOI:** 10.1016/j.media.2024.103386

**Published:** 2024-11-30

**Authors:** Sam Coveney, Maryam Afzali, Lars Mueller, Irvin Teh, Arka Das, Erica Dall’Armellina, Filip Szczepankiewicz, Derek K. Jones, Jurgen E. Schneider

**Affiliations:** a Biomedical Imaging Science Department, Leeds Institute of Cardiovascular and Metabolic Medicine, University of Leeds, Leeds, United Kingdom; b Medical Radiation Physics, Clinical Sciences Lund, Lund University, Lund, Sweden; c Cardiff University Brain Research Imaging Centre (CUBRIC), School of Psychology, Cardiff University, Cardiff, United Kingdom

**Keywords:** Diffusion tensor imaging, Magnetic resonance imaging, Cardiac, Outlier detection, Robust estimation, M-estimator, 41A05, 41A10, 65D05, 65D17

## Abstract

Cardiac diffusion tensor imaging (cDTI) is highly prone to image corruption, yet robust-fitting methods are rarely used. Single voxel outlier detection (SVOD) can overlook corruptions that are visually obvious, perhaps causing reluctance to replace whole-image shot-rejection (SR) despite its own deficiencies. SVOD’s deficiencies may be relatively unimportant: corrupted signals that are not statistical outliers may not be detrimental. Multiple voxel outlier detection (MVOD), using a local myocardial neighbourhood, may overcome the shared deficiencies of SR and SVOD for cDTI while keeping the benefits of both. Here, robust fitting methods using M-estimators are derived for both non-linear least squares and weighted least squares fitting, and outlier detection is applied using (i) SVOD; and (ii) SVOD and MVOD. These methods, along with non-robust fitting with/without SR, are applied to cDTI datasets from healthy volunteers and hypertrophic cardiomyopathy patients. Robust fitting methods produce larger group differences with more statistical significance for MD, FA, and E2A, versus non-robust methods, with MVOD giving the largest group differences for MD and FA. Visual analysis demonstrates the superiority of robust-fitting methods over SR, especially when it is difficult to partition the images into good and bad sets. Synthetic experiments confirm that MVOD gives lower root-mean-square-error than SVOD.

## 1 Introduction

Cardiac diffusion tensor imaging (cDTI) is a non-invasive, contrast agent and ionizing-radiation free method for characterizing myocardial microstructure, based on the (anisotropic) random motion of water molecules (Reese et al., 1995; Scollan et al., 1998). Diffusion models can be fitted to images obtained with sensitization to diffusion in different directions and amplitudes. A diffusion tensor model is commonly fitted for each voxel (Basser et al., 1994), from which quantities such as mean diffusivity (MD) and fractional anisotropy (FA) can be obtained (Basser and Pierpaoli, 1996), as well as various biomarkers of tissue orientation and organization such as the primary cardiomyocyte direction and laminar-aggregate orientation characterized by the ‘sheetlet’ angle E2A (Kung et al., 2011; Ferreira et al., 2014; Nielles-Vallespin et al., 2017). These metrics have been shown to vary between different pathologies and healthy states, and therefore give insight into how tissue structure and organization are affected by myocardial disease (Ferreira et al., 2014; Abdullah et al., 2014; von Deuster et al., 2016; Khalique et al., 2018; Ariga et al., 2019; Gotschy et al., 2020; Nielles-Vallespin et al., 2020; Das et al., 2021; Rahman et al., 2022; Eder et al., 2022; Das et al., 2023; Joy et al., 2023).

Cardiac DTI is challenging compared to DTI of other organs, mainly due to cardiac and respiratory motion, which is several orders of magnitude larger than the motion arising from the diffusing water molecules. Sequence design and bespoke motion compensation schemes have advanced significantly (Stoeck et al., 2016), but repeated imaging with the same contrast is commonly performed in cDTI to provide data redundancy and compensate for relatively low signal-to-noise ratio (SNR). Respiratory motion is typically corrected for through image registration in post-processing. However, besides thermal noise there generally remains additional structured variation between images that nominally have the same contrast. There are generally small variations in cardiac phase and heart position relative to the imaging plane. Additionally, motion is expected to modulate image intensities via differences in diffusion gradients relative to tissue (Leemans and Jones, 2009), *B*_0_-field inhomogeneities (Reeder et al., 1998; Verma and Cohen-Adad, 2014), coil sensitivities (Faraji-Dana et al., 2016), and interpolation effects from image registration (Vishnevskiy et al., 2015). These variations are not specifically modelled, and are sometimes referred to as ‘physiological noise’ as opposed to ‘thermal noise’.

Corrupted images are also a common problem in cDTI: signal drop-out due to susceptibility differences is common, image registration can fail, and physiological noise can be too severe. In cDTI, these ‘outlier’ images are usually identified by manual inspection and removed before fitting diffusion models, although there have been some attempts to automate this (Ferreira et al., 2020; Coveney et al., 2023). However, there is a wealth of literature for handling outlier data in diffusion MRI of the brain using robust fitting and identification of corrupted images. Yet, these are not commonly applied in cDTI. We therefore briefly review various approaches to deal with outliers.

*Single-voxel outlier detection* (SVOD) methods use iterative fitting to implement a robust estimator that is insensitive to outlier data (Mangin et al., 2002), usually followed by outlier removal and refitting with a non-robust estimator, such as in the RESTORE/iRESTORE algorithm (Chang et al., 2005, 2012). Each voxel is processed independently. Various choices of robust estimators are possible (Maximov et al., 2011), and robust estimators have been developed for the linearized formulation (fitting models to the log-signal) of DTI (Collier et al., 2015) and diffusion kurtosis modelling (DKI) (Tax et al., 2015). ‘Model-free’ outlier correction for DTI signals have also been developed (Niethammer et al., 2007). Iterative fitting without robust estimators has been used with outlier detection at each iteration (Pannek et al., 2012). RESTORE has been applied to physiological noise in the brain (Walker et al., 2011).

*Multiple-voxel outlier detection* (MVOD) leverages the information from multiple voxels to detect outliers, which are then discarded before fitting. The correlation of images between successive slices (Liu et al., 2010), between each image and a reference image (Li et al., 2014; Ferreira et al., 2020) as well as similarity between automatic segmentation masks (Ferreira et al., 2022) can be used to detect outliers. Texture features (Zhou et al., 2011), intensity histograms (Scelfo et al., 2012) and spherical histograms of principle directions (Farzinfar et al., 2013) have also been applied for outlier detection. We have previously used the mean-squared error between observed and model-predicted images to identify outliers in *cardiac* DTI (Coveney et al., 2023). It is also possible to incorporate measures of image quality into fitting weights to account for the variable quality of images. In Zwiers (2010), weights are based on the square of the mean error of the model fit across each image. In Li et al. (2013), weights depend on both voxel-wise residuals and “inter-slice intensity discontinuity”. The intensity variance of images has been used for weighting and “slice-wise outlier detection” (Sairanen et al., 2018).

**Fig. 1:**
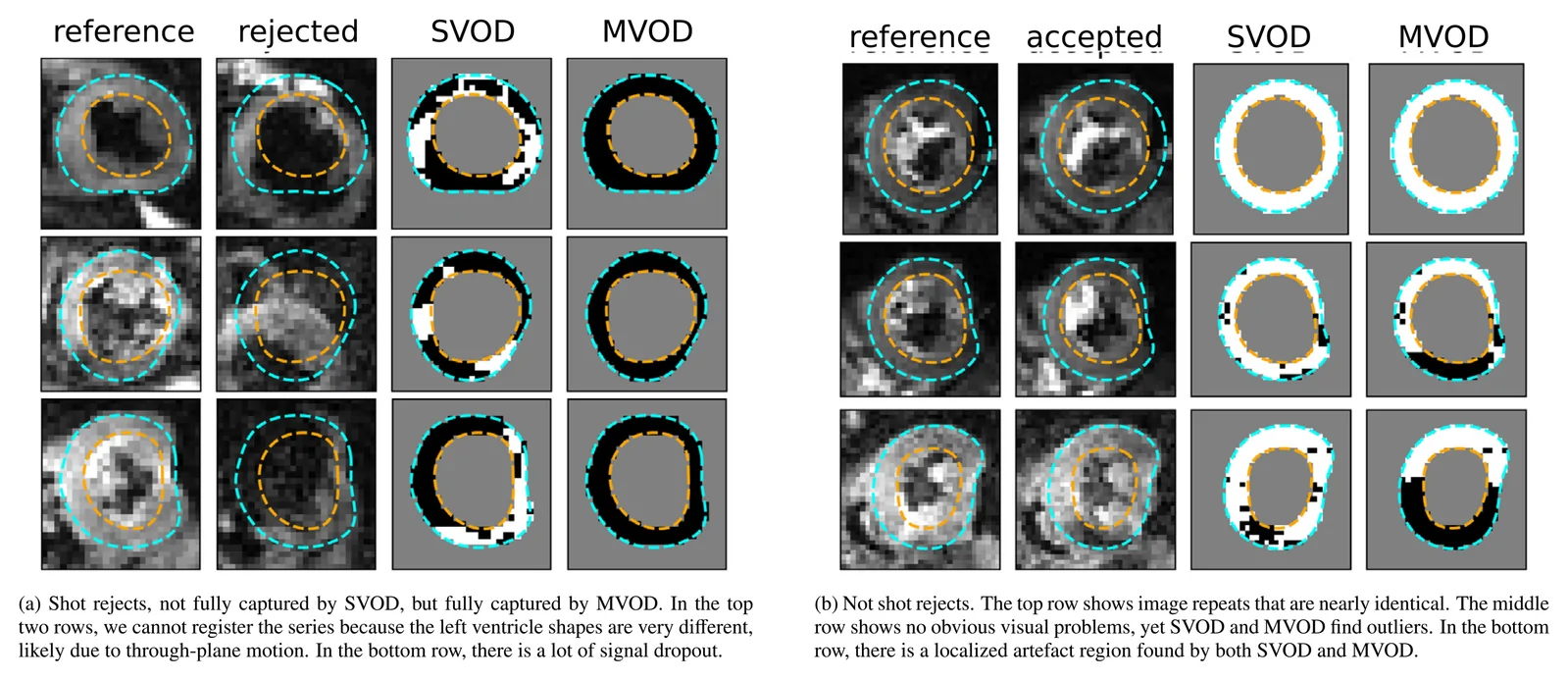
Examples from 6 datasets (1 per row in each sub-figure) (a) examples where shot-rejection has (correctly) identified a corrupted image, but SVOD has not identified all myocardial voxels in the corrupted image; (b) examples where shot-rejection has *not* identified a corrupted image. The ‘reference’ image shows a typical ‘good image’, while the ‘accepted’ or ‘rejected’ image columns show a different image with the same diffusion weighting for the same subject. The myocardial segmentation is also shown. For SVOD and MVOD columns, black/white voxels indicate outlier/non-outlier signals respectively.

In cDTI, images severely corrupted by artefacts are commonly removed entirely from a dataset before fitting models. This is commonly referred to as ‘shot-rejection’ (SR). Furthermore, the (lack of) literature suggests that robust fitting methods are rarely if ever used in cDTI. Manual identification of corrupted images – while common – is subjective and time-consuming, and the paradigm of perfectly separating images into good and bad sets may be flawed due to physiological noise. Our current work presented here was motivated by the observation that many ‘incorrect’ signals in obviously corrupted images (e.g. a large difference in cardiac phase; a complete failure of image registration; etc.) are not identified as outliers by SVOD methods. Fig. 1(a) shows some example ‘bad images’ (rejected) alongside equivalent ‘good images’ (reference) with the same diffusion weighting. In this case, our SR method (see Appendix A) identified these images for removal, and these corruptions seem visually obvious. Nonetheless, the maps indicating robust signals (i.e. non-outlier signals) for the ‘bad images’ are shown for the SVOD and MVOD algorithms introduced later in this paper (Sections 2.2 and 2.3 respectively). SVOD, which considers data across all images but for each voxel independently, fails to classify many of the incorrect signals as outliers, while the proposed MVOD method classifies all signals as outliers. Fig. 1(b) is similar, except the second column shows images that were not marked for SR, and we feel that visual inspection would not have indicated the need for SR (with the possible exception of the last row of images, in which case an entire image would need removing for the sake of a localized corruption). The first row shows image repeats that are nearly identical, and neither SVOD nor MVOD detects non-robust signals. However, for rows two and three, SVOD detects non-robust signals even though the images were not rejected by SR. MVOD detects more non-robust signals, and certainly seems to cover more of the artefact area in the last row. Fig. 1 is included here to illustrate the motivation behind the current work. Importantly, it is not immediately obvious whether a failure to identify all ‘incorrect signals’ with SVOD (or even with MVOD) is actually important for the problem at hand, i.e. robust estimation of diffusion tensor model parameters. Identification of outliers in robust fitting algorithms is only an incidental goal: if an incorrect signal is not statistically identifiable as an outlier *even when using robust fitting*, it may not have an adverse effect on parameter estimation. However, to the best of our knowledge, a systematic investigation of this problem has never been performed despite image corruption being a fundamental and common problem in cDTI.

In this paper, we present iteratively reweighted least squares (IRLS) methods using robust M-estimator weights together with SVOD and MVOD techniques. These methods are formulated similarly to previous algorithms in the literature, and can be applied to both the non-linear (i.e. fit to signal) and linearized (i.e. fit to log-signal) versions of the fitting problem. We apply these methods, as well as non-robust fitting with and without shot rejection, to a dataset of healthy volunteers (HV) and hypertrophic cardiomyopathy (HCM) patients (Das et al., 2022). We also perform synthetic experiments using artificially corrupted datasets to compare SVOD against MVOD. Our results indicate that robust fitting methods outperform shot-rejection, generating larger group differences with more statistical significance. MVOD produces larger group differences than SVOD for MD and FA, and the synthetic experiments indicate that MVOD is better able to recover diffusion metrics from corrupted data than SVOD. Nonetheless, the benefits of MVOD over SVOD (as applied on the original datasets) seem minor, suggesting that the deficiencies of SVOD in identifying all incorrect signals may not be important in practice. Therefore, robust estimation with either SVOD or MVOD should be capable of entirely replacing shot-rejection in cDTI.

## 2 Methods

DTI data are a series of *n* images *i* = 1 … *n* obtained with diffusion weightings *b*_*i*_ and directions (unit vectors) **g**_**i**_ = (*g*_*i,x*_, *g*_*i,y*_, *g*_*i,z*_). Considering a single voxel, the noisy signal *y*_*i*_ observed in image *i* can be related to the signal model *f* (***θ***, *b*_*i*_, **g**_**i**_), with parameters ***θ*** and the error *ϵ*_*i*_ as:

(1) $$y_{i}=f\left(\theta ,b_{i},g_{i}\right)+\epsilon_{i}$$

We focus on the diffusion tensor model of the signal:

(2) $$f\left(\theta ,b_{i},g_{i}\right)=S_{0}\exp\left(-b_{i}\;g_{i}\;\text{D}\;g_{i}^{T}\right)=\exp\left(x_{i}\theta^{T}\right)$$

where $x_{i}=(\;1,-b_{i}g_{i,x}^{2},-2b_{i}g_{i,x}g_{i,y},-2b_{i}g_{i,x}g_{i,z},-b_{i}g_{i,y}^{2},-2b_{i}g_{i,y}g_{i,z},-b_{i}g_{i,z}^{2}\;),$ and model parameters *θ* = (log(*S*_0_), *D*_*xx*_, *D*_*xy*_, *D*_*xz*_, *D*_*yy*_, *D*_*yz*_, *D*_*zz*_). The methods presented here can apply to other models, such as the diffusion kurtosis imaging (DKI) model. We denote the standard deviation of the Gaussian noise in the original complex images as *σ*. We consider magnitude images, for which the noise distribution can be complicated (St-Jean et al., 2020); we assume Rician distributed error for simplicity (Cárdenas-Blanco et al., 2008), and note that *ϵ*_*i*_ results from both the Gaussian noise in the complex images and the magnitude operation.

Estimators for parameters ***θ*** are defined in terms of an objective function. For convenience, we write *f*_*i*_ ∶= *f* (***θ***, *b*_*i*_, **g**_**i**_). Defining the fitting residuals *r*_*i*_ ∶= *y*_*i*_ − *f*_*i*_, the non-linear least squares (NLLS) estimator is given by

(3) $$\hat{\theta }=\text{argmin}\sum_{i=1}^{n}{\left(\frac{r_{i}}{\sigma }\right)}^{2}$$

(4) $$=\text{argmin}\sum_{i=1}^{n}\left(\frac{1}{\sigma^{2}}\right)r_{i}^{2}$$

Eqs. (1) and (2) can be cast as a linear problem:

(5) $$\log\left(y_{i}\right)=\log\left(f_{i}\right)+\epsilon_{i}^{*}$$

(6) $$=x_{i}\theta^{T}+\epsilon_{i}^{*}$$

For sufficiently high SNR, we can approximate the standard deviation of the error $\epsilon_{i}^{*}$ as $\sigma_{i}^{*}=\sigma /f_{i}$ where *f*_*i*_ is the predicted signal (Salvador et al., 2005). Defining the residuals of the logs as *u* ∶= log(*y*) − log(*f*), the weighted least squares (WLS) estimator is given by:

(7) $$\hat{\theta }=\text{argmin}\sum_{i=1}^{n}{\left(\frac{f_{i}u_{i}}{\sigma }\right)}^{2}$$

(8) $$=\text{argmin}\sum_{i=1}^{n}\left(\frac{f_{i}^{2}}{\sigma^{2}}\right)u_{i}^{2}$$

Since *f*_*i*_ is not known in advance of fitting the parameters, it can be replaced with the measured signal *y*_*i*_ or with an estimate of *f*_*i*_ obtained from ordinary (non-weighted) least squares. We use the latter in this work. Eq. (6) is linear in the parameters, so $\hat{\theta }$ is given analytically by the weighted least squares formula.

### 2.1 Robust weighting with M-estimators

Least-squares estimators per se are not robust against outliers, so corrupted data can have a severe effect on the model fit. Robust fitting methods such as M-estimators replace the squared operation with a subquadratic function *ρ*(*R*_*i*_), such that $\rho \left(R_{i}\right)\leq R_{i}^{2},$ giving estimators of the form:

(9) $$\hat{\theta }=\text{argmin}\sum_{i=1}^{n}\rho (R_{i})$$

It can be beneficial to recast the M-estimator problem as an iteratively reweighted least squares (IRLS) problem by deriving a set of weights from *ρ*. This has the advantage that algorithms designed for least-squares estimation, such as the Levenberg–Marquardt algorithm for non-linear fitting or the standard weighted least squares estimator for linear fitting, can be utilized. The parameter vector ***θ*** that solves Eq. (9) also solves:

(10) $$\sum_{i=1}^{n}\frac{\partial \rho }{\partial \theta_{j}}=\sum_{i=1}^{n}\psi \left(R_{i}\right)\frac{\partial R_{i}}{\partial \theta_{j}}=0,\;\text{for}\;j=1,\ldots ,m$$

where *j* indexes into *m* parameters, and *ψ*(*R*_*i*_) = *d ρ*(*R*_*i*_)/d*R*_*i*_ is called the *influence function*. If we define *w*(*R*_*i*_) = *ψ*(*R*_*i*_)/*R*_*i*_ then we can write

(11) $$\sum_{i=1}^{n}w\left(R_{i}\right)R_{i}\frac{\partial R_{i}}{\partial \theta_{j}}=0,\;\text{for}\;j=1,\ldots ,m$$

which is the solution to the IRLS problem:

(12) $$\hat{\theta }=\text{argmin}\frac{1}{2}\sum_{i=1}^{n}w(R_{i}^{k-1})R_{i}^{2}$$

where *k* ≥ 1 is the iteration number, and $R_{i}^{k-1}$ are the residuals from the previous iteration (for *k* = 1, the residuals from the corresponding non-robust least squares problem can be used).

To derive the robust estimators for replacing the least squares estimators in Eqs. (4) and (8), we substitute *R*_*i*_ in *ρ*(*R*_*i*_) with the corresponding quantity. We use the Geman-McClure M-estimator (GMM estimator) here $\rho \left(R_{i}\right)=R_{i}^{2}/(1+R_{i}^{2}).$ For the NLLS problem *R*_*i*_ = *r*_*i*_/*σ*, we obtain:

(13) $$\hat{\theta }=\text{argmin}\sum_{i=1}^{n}{\left(\frac{\sigma^{2}}{{\left(\sigma^{2}+r_{i}^{2}\right)}^{2}}\right)}^{k-1}r_{i}^{2}$$

and for the linear problem with *R*_*i*_ = *f*_*i*_*u*_*i*_/*σ* we obtain:

(14) $$\hat{\theta }=\text{argmin}\sum_{i=1}^{n}{\left(\frac{{\left(\sigma /f_{i}\right)}^{2}}{{\left({\left(\sigma /f_{i}\right)}^{2}+u_{i}^{2}\right)}^{2}}\right)}^{k-1}u_{i}^{2}$$

The weights for the squared-residuals $(\;r_{i}^{2}\;\text{or}\;u_{i}^{2}\;)\;$ can be inferred from comparing Eqs. (13) and (14) with Eq. (12^*i*^) (the constant factor 1/2 can be neglected). Algorithm 1 shows pseudo-code for our IRLS procedure. We have presented the derivations above for two important reasons.

We have presented the derivations above for two important reasons. Firstly, many DTI papers and software packages that purport to use the Geman-McClure M-estimator fail to use the proper weights, which has been noted elsewhere (Tax et al., 2015). Secondly, Eq. (14) is different from the robust weights suggested for the WLS problem in both Tax et al. (2015) and Zwiers (2010), but the same result as Collier et al. (2015). There are many variations in the literature for robust

Algorithm 1 **Iteratively Reweighted Least Squares for DWI models**

**FUNCTION** robustweightsWLS(…, k, K)

   **if** k < K - 1 **then**

     **return** robust WLS weights

   **if** k == K - 1 **then**

     test for robustness

     **return** 0 or 1

   **if** k == K **then**

     use last robustness condition

     **return** 0 or predictedsignal^2^

**FUNCTION** robustweightsNLLS(…, k, K)

   **if** k < K **then**

     **return** robust NLLS weights

   **if** k == K **then**

     test for robustness

     **return** 0 or 1

**Input**: signals, bvals, bvecs, K, FITTYPE

**Output**: parameters, robustmask

**for** k in 1…K **do**

   **if** k == 1 **then**

     solve non-robust problem

   **if** k > 1 **then**

     **w** ← robustweightsFITTYPE(…, k, K)

     solve robustly weighted problem

DTI fitting algorithms, including: whether to always/conditionally use robust fitting; what this condition is; how to identify outliers; how to test IRLS convergence; use of different estimators; fitting with NLLS or WLS; etc. We give additional details for our algorithm here, making sure to keep consistency between the NLLS and WLS methods.

Like several previous works, we utilize the Median Absolute Deviation (MAD) for estimating the noise level $\hat{\sigma }$ required for Eqs (13) and (14). For the non-linear problem (as in Chang et al., 2012):

(15) $$\hat{\sigma }\approx 1.4826\frac{n}{n-m}\times \text{MED}\left(\left|r_{i}-\text{MED}\left(r_{i}\right)\right|\right)$$

and for the linear problem (as in Collier et al., 2015):

(16) $$\hat{\sigma }\approx 1.4826\frac{n}{n-m}\times \text{MED}\left(\left|f_{i}u_{i}-\text{MED}\left(f_{i}u_{i}\right)\right|\right)$$

Goodness of fit tests for using robust fitting (as opposed to accepting the initial non-robust estimate) include analysing residuals with a 3-sigma test (Chang et al., 2005) and a chi-squared test (Chang et al., 2012; Collier et al., 2015). However, robust estimation is used for outlier detection precisely because it is difficult to identify outliers from an initial non-robust fit, therefore we always use robust fitting (as also done by Tax et al. (2015) for fitting diffusion MRI data of the brain). Initially, we tested for convergence of IRLS by checking if the norm of the parameter vector changes by less than 0.1 percent of the value from the last iteration, stopping at 10 iterations (this limit was rarely reached). However, for simplicity we fixed the number of iterations to 10. We implemented our IRLS algorithms in DIPY (Garyfallidis et al., 2014).

### 2.2 Single-voxel outlier detection

After robust fitting, outliers are usually identified based on a 3-sigma test. In Collier et al. (2015), negative residuals are tested with $\left|u_{i}\right|>3\hat{\sigma }/f_{i}$ and positive residuals with $\left|r_{i}\right|>3\hat{\sigma },$ but in our synthetic experiments we found that this ‘two-eyes’ approach caused underestimation of MD for low SNR. For simplicity and equivalence, we applied the outlier test $\left|r_{i}\right|>3\hat{\sigma }$ for both positive and negative residuals for both NLLS and WLS. Note that residuals (unlike errors) are neither independent nor identically distributed. Thus, residuals should be corrected at the outlier-detection stage by dividing them by the factor $\sqrt{1-h_{ii}},$ where the leverage *h*_*ii*_ is the diagonal of the ‘hat’ (projection) matrix that maps the observed signals to the predicted signals. We use the hat matrix from the initial OLS/final WLS fit for the NLLS/WLS problem respectively, similar to Collier et al. (2015); see Appendix C for details. Admittedly, we saw little evidence that adjusting residuals in this way made a substantial difference, but we included this adjustment for thoroughness. After outlier identification, we perform a final non-robust fit without outliers, using the original least-squares estimators.

### 2.3 Multiple-voxel outlier detection

In a local spatial neighbourhood, the mean residual is:

(17) $$\mu_{il}=\frac{1}{N_{\upsilon }}\underset{j\in N(l)}{\sum }r_{ij}$$

and the root mean squared residual is:

(18) $$\sigma_{il}=\sqrt{\frac{1}{N_{\upsilon }}\underset{j\in N(l)}{\sum }r_{ij}^{2}}$$

where *l* indexes voxels, and *𝒩* (*l*) is the set of *N*_*v*_ voxel indices within a local neighbourhood of voxel *l*. The neighbourhood is restricted to the myocardial mask within each image and defined by a cut-off distance (details on defining a myocardial mask can be found in Appendix B). Note that the residuals *r*_*ij*_ correspond to the signal (not the log-signal) for both NLLS and WLS, and these residuals are adjusted for leverages as explained for SVOD. We used a simple MVOD condition for both the mean residual and the root mean squared residual: if *t*_*il*_ > 3 × 1.4826 × MED_*i*_ *t*_*il*_ − MED_*i*_(*t*_*il*_), where *t*_*il*_ is either *µ*_*il*_ or *σ*_*il*_, then signal *i* in voxel *l* is an outlier according to MVOD. If the spatial neighbourhood is large enough to include all myocardial voxels in the image, then *t*_*il*_ will be the same for all *l* voxels (in which case, all voxels in each image *i* will either be rejected or accepted). MVOD is applied in addition to SVOD.

Occasionally in our synthetic experiments (see Section 2.5) for voxels with very high SNR on the septum, robust estimation via IRLS led to different distributions of *µ*_*il*_ or *σ*_*il*_ for different b-shells in some voxels, such that the MVOD conditions could remove too many low-B images making the resulting design matrix rank-deficient. This sort of problem can occur with SVOD as well (Chang et al., 2012), although we never observed this. It is possible that this issue relates to IRLS as a means of implementing robust estimation (Baselga et al., 2021). Since this only affected a few voxels in our synthetic experiments, we opted to use singular-value-decomposition to check if the smallest eigenvalue of the design matrix (with SVOD and MVOD outliers removed), was near zero; if it was, then we did not apply MVOD in addition to SVOD. We never observed this problem outside of the synthetic experiments.

### 2.4 Datasets

We utilized data from 11 healthy volunteers and 16 HCM patients from a previous study (Das et al., 2022). Each full data set comprised b-values of 100 s/mm^2^ (3 DW directions, 12 repetitions), and 450 s/mm^2^ (30 DW directions, 6 repetitions). After image registration using SimpleElastiX (Marstal et al., 2016), we used shot-rejection as described in Appendix A to attempt to identify corrupted images. We fit tensors to these cases using either NLLS or WLS, and segmented the myocardium as described in Appendix B.

To these datasets, we applied non-robust estimation (NLLS or WLS), non-robust estimation with shot-rejection (NLLS_SR or WLS_SR), robust-estimation with SVOD (RNLLS or RWLS), robust estimation with SVOD and MVOD (RNLLS_MV and RWLS_MV) using a cut-off distance of 10 voxels. We calculated the mean values of MD, FA, and absolute E2A according to the AHA segments, within each short-axis slice, and globally (all slices), excluding regions of artefact (see Appendix B) since these regions do not represent tissue properties. We also calculate Helix Angle (HA).

We calculated the difference of the means and medians of the groups for MD, FA, and absolute E2A, and also applied the Mann–Whitney U-test to test for significance. We also calculated the root-mean-squared-error of the fits (based on the predicted signal of the fit versus the observed signal) excluding the identified outliers in the case of robust fitting methods, and excluding the rejected images in the case of shot-rejection.

### 2.5 Synthetic experiments

We ran synthetic experiments on the datasets to evaluate the different fitting methods. In place of a true ‘ground truth’, we used the fitting results of robust-estimation with SVOD for WLS (i.e. RWLS) as a ‘synthetic ground-truth’ for each subject from which noiseless images could be generated by prediction through the signal model. Corruptions followed by Rician noise were then applied. The following types of synthetic corruptions were applied to a percentage of randomly selected images from the synthetic ground-truth in each slice: **deregistration**: corrupt image by 2 voxel translation in random direction; **b-vector swap**: replace image with prediction from a different diffusion direction **g**, generated from a random 90 degree rotation of the true diffusion direction for that image; **random field**: corrupt image by multiplying it by the exponential transform of a sample from a spatially-correlated Gaussian random field *g*(*z*) ~ *N* (0, *k*(**z**_**l**_, **z**_**m**_)), with covariance kernel *k*(**z**_**l**_, **z**_**m**_) = *A* exp(−|**z**_**l**_ − **z**_**m**_ |^2^/*L*^2^), where **z** are voxel coordinates (not spatial coordinates), length-scale *L* = 15, and amplitude *A* = 1/3 (the resulting sample oscillates around 1, mostly between 0.5 and 2.0). Rician error was then applied to all images: defining SNR as the average *S*_0_ over myocardial voxels divided by the standard deviation *σ* of complex Gaussian noise, we used a defined SNR to determine *σ*, and used this to generate Rician error. For our experiments, we used corruption percentages of 10%, 25%, and 40%. We tested SNR of 40, 25, 10.

For each combination of corruption type, corruption percentage, and SNR, we created 10 synthetic datasets per subject, to which non-robust fitting (NLLS/WLS), robust fitting with SVOD (RNLLS/RWLS), and robust fitting with MVOD (RNLLS_MV_X /RWLS_MV_X) were applied: for MVOD methods, we tested myocardial neighbourhoods defined by a distance of X = 5 and X = 10 voxels, and also a neighbourhood including X = all voxels in the slice. After each fit, we calculate global MD, FA, and absolute E2A, such that there is a range of fitting results for each combination and for each fitting method, from which the root-mean-squared-error could be calculated. Importantly, for low SNR the average result of fitting with NLLS or WLS *without corrupted images* can be biased i.e. not equal to the synthetic ground-truth. We therefore calculated the root-mean-square-error with respect to the average (over the 10 synthetic datasets) of results of NLLS or WLS on the non-corrupted images, since if robust fitting could identify all corrupted signals then this definition of RMSE would be zero. This isolates the effects of robust fitting from the effects of metric bias caused by noise. For each combination of corruptions, we applied a Wilcoxon signed-rank test on the RMSE between the SVOD method (RNLLS/RWLS) and each of the MVOD methods (RNLLS_MV_X/RWLS_MV_X), testing for significance using *p* < 0.05/6 in order to compensate for 6 tests being performed (3 for NLLS-type fits, 3 for WLS-type fits) for each corruption combination.

## 3 Results

### 3.1 Application to datasets

**Fig. 2:**
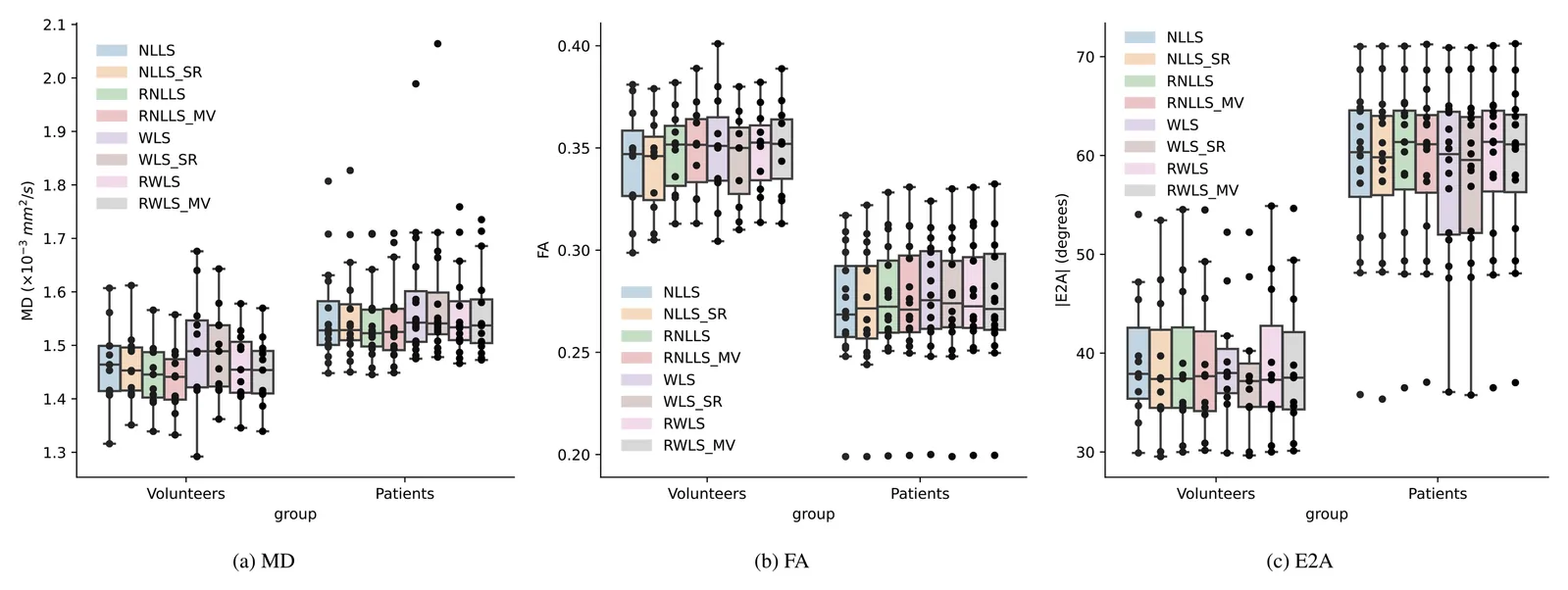
Diffusion measures MD (×10^−3^ mm^2^ /s), FA, and absolute E2A (degrees) for volunteers and HCM patients for different fitting methods.

Fig. 2 shows global MD, FA, and absolute E2A, as fit to the original datasets, for the methods explained in Section 2.4. Table 1 shows the group differences of the mean and median, and the *p*-value, for the methods in Fig. 2. Table 1 shows that the group differences between Volunteers and HCM Patients was largest for MVOD methods for both MD and FA, and the significance was also higher (the *p*-value was lower). The SVOD methods showed the second largest difference between the groups for MD and FA, except for RWLS where shot-rejection (WLS_SR) showed a slightly larger difference for mean MD (although the values are nearly identical) — note that difference of medians was nontheless much bigger for RWLS MD than for WLS_SR MD, and RWLS had nearly 6 times decrease in *p*-value compared to WLS_SR. It is visually clear that robust-fitting methods seem to reduce the spread of metric values in the healthy volunteers, especially for WLS methods. Importantly for WLS, the *p*-value for MD does not pass the standard significance test of *p* < 0.05 at least without shot rejection, and the robust-fitting methods are much more significant still. For E2A, either SVOD or MVOD gave the largest group differences — there are several ties in the *p*-value due to the nature of the Wilcoxon signed-rank test. Care should be taken to interpret the box-plots (depending on whether points are classed as ‘fliers’, the box and whiskers will be drawn differently, which can lead to discontinuous changes in the box sizes). For WLS and WLS_SR methods, the box plot for volunteer E2A seems narrower than for the robust methods, but in fact this is because the robust methods do not produce as many flier (out-lying) values of E2A, so in this respect the ‘spread’ is reduced for robust methods.

**Fig. 3:**
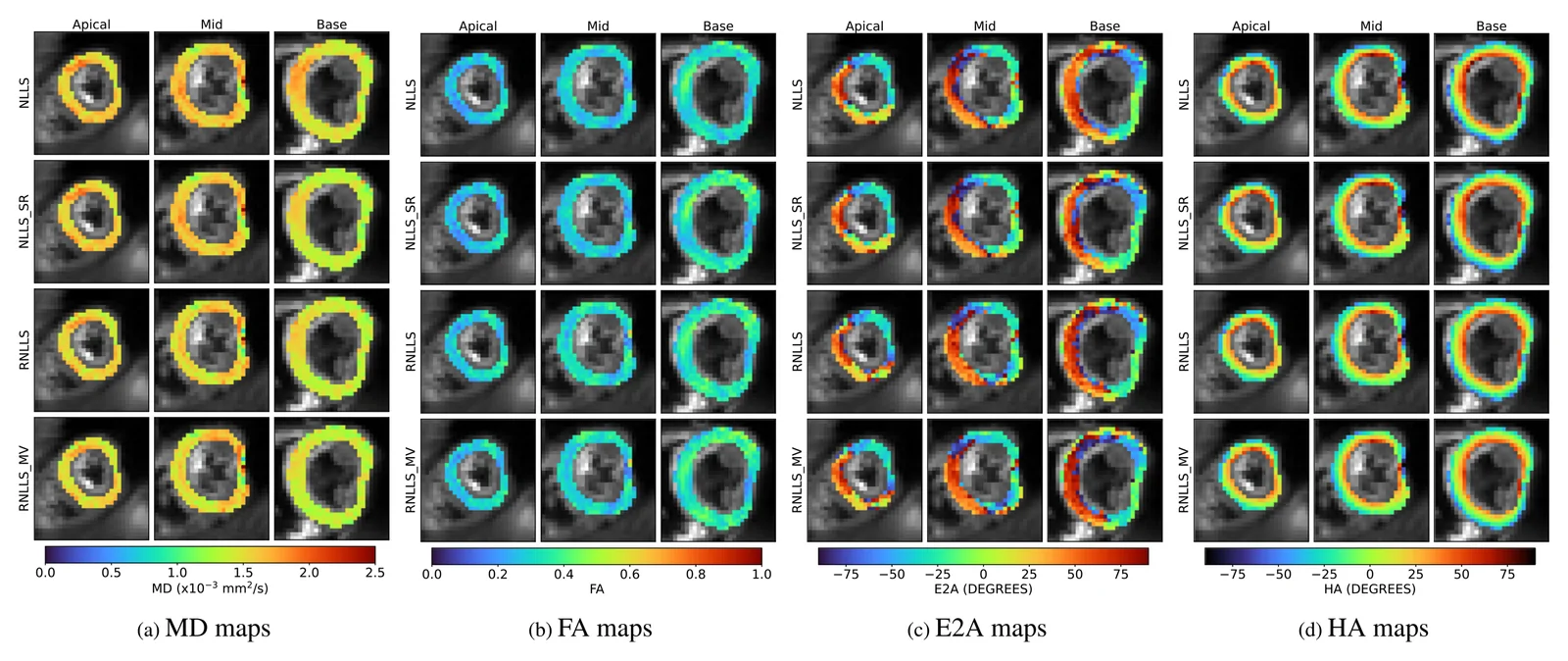
Example healthy volunteer (highest MD from non-robust WLS fitting in the HV group of Fig. 2). First row: non-robust NLLS; Second row: non-robust NLLS after shot-rejection; Third row: robust NLLS with SVOD; Fourth row: robust NLLS with SVOD and MVOD (10 voxel neighbourhood).

**Fig. 4:**
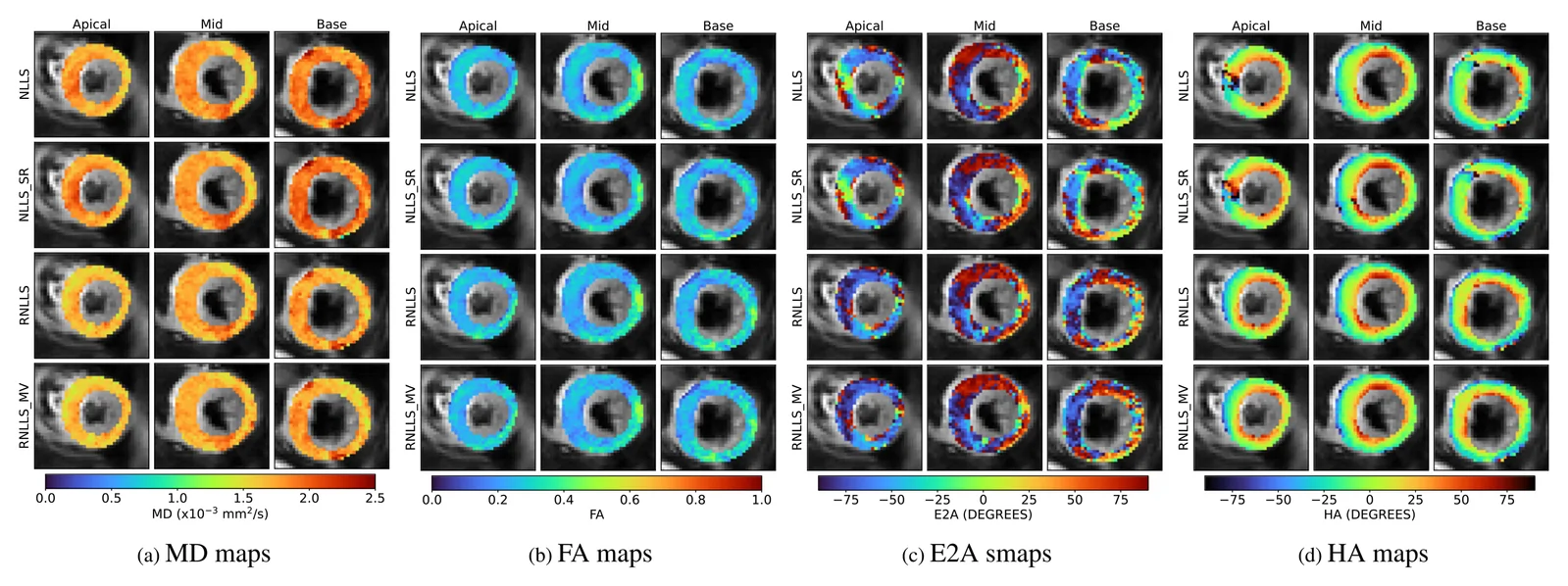
Example HCM patient (highest MD from non-robust WLS fitting in the HCM group of Fig. 2). First row: non-robust NLLS; Second row: non-robust NLLS after shot-rejection; Third row: robust NLLS with SVOD; Fourth row: robust NLLS with SVOD and MVOD (10 voxel neighbourhood).

Figs. 3 and 4 show MD, FA, E2A and HA (helix angle) maps for an example volunteer and HCM patient respectively. These examples were the subjects with the largest MD values for the non-robust WLS methods. Furthermore, even attempts at manual shot-rejection were extremely difficult in these cases, since it was difficult to separate good from bad images: even the better images appeared to contain large shot-to-shot variations and corruptions at the sub-image level. Note that, we have shown all the myocardium in Figs. 3 and 4, although regions of distortion and isolated artefacts were excluded from global metric calculations, as explained in Appendix B.

Fig. 3 shows a marked decrease in MD across the myocardium for robust fitting compared to non-robust fitting (with or without shot-rejection), especially around the septum. This decrease is slightly larger for MVOD than SVOD, especially in the basal slice. The effects on FA are equivalent but in the opposite direction (an increase). The transmural variation of HA from right-handed (red) to left-handed (blue) is also superior in MVOD, with the blue fibres in the bottom left of the mid-slice only convincingly recovered with MVOD. Additionally, SVOD and MVOD both show significantly more convincing transmural variation from red (right-handed) to green (circumferential) to blue (left-handed) in the septum of the basal slice, where the non-robust (with or without SR) methods show an extended region of red in the same area.

Fig. 4 shows a strong increase in (magnitude of) E2A in the septum for robust methods, particularly in the apical and basal slices. These changes are also accompanied by a complete recovery of HA by robust fitting methods, whereas there are obvious corruptions of HA in both non-robust methods (including shot-rejection). Especially important is the recovery of right-handed (red) HA in the septum of the basal slice, for both robust methods, whereas these right-handed fibres appear to be missing for not-robust methods. SVOD and MVOD appear to decrease MD substantially, moreso for MVOD than for SVOD.

We reviewed differences in fitting methods for every subject by eye. Overall, the visual differences in diffusion measure maps between SVOD and MVOD seemed quite minor for most subjects. This seems consistent with Fig. 2 and Table 1: while MVOD modifies the results further in the ‘same direction’ in which SVOD improves upon shot-rejection, the improvement upon SVOD would appear relatively minor in comparison to the improvement that SVOD makes over shot-rejection.

**Fig. 5:**
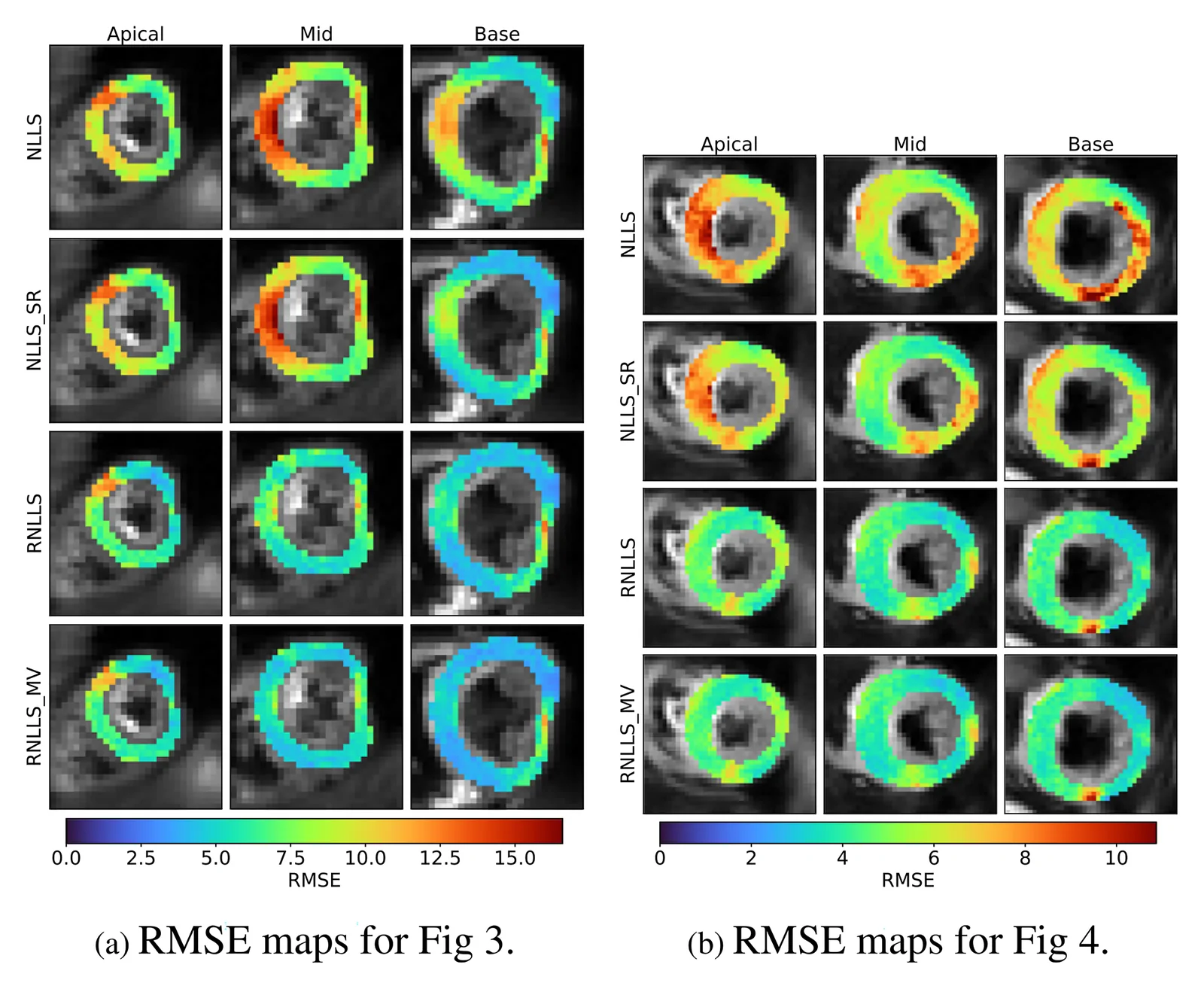
RMSE of fitting residuals, excluding identified outliers or excluded images (as applied). RMSE is lower and more uniform for robust fitting methods, being lower for MVOD than SVOD, with remaining high RMSE regions seeming to indicate patches of persistent artefact across the image series.

The RMSE of the predicted versus observed signals, corresponding to Figs. 3 and 4, are shown in Fig. 5. What is especially clear is that while shot-rejection can reduce the RMSE of the fit to some degree, the robust-estimation with SVOD and MVOD do substantially better, leaving a nearly uniform (and lower) RMSE except in regions of isolated artefact. Note that, after robust-fitting, these artefact regions are extremely easy to identify from RMSE, suggesting that robust-fitting is able to turn RMSE into a useful metric for determining the quality of the fit in a way that becomes independant of outliers and corruption in some images, therefore leaving only the effects of artefacts that permeate the entire image series. Even after robust fitting, there are some regions of elevated MD in Figs. 3 and 4 that remain, but these correspond to significantly higher RMSE than the rest of the myocardium, suggesting that these are likely to be artefacts not real features. Note that MVOD has lower and more uniform RMSE than SVOD.

### 3.2 Synthetic experiments

**Fig. 6:**
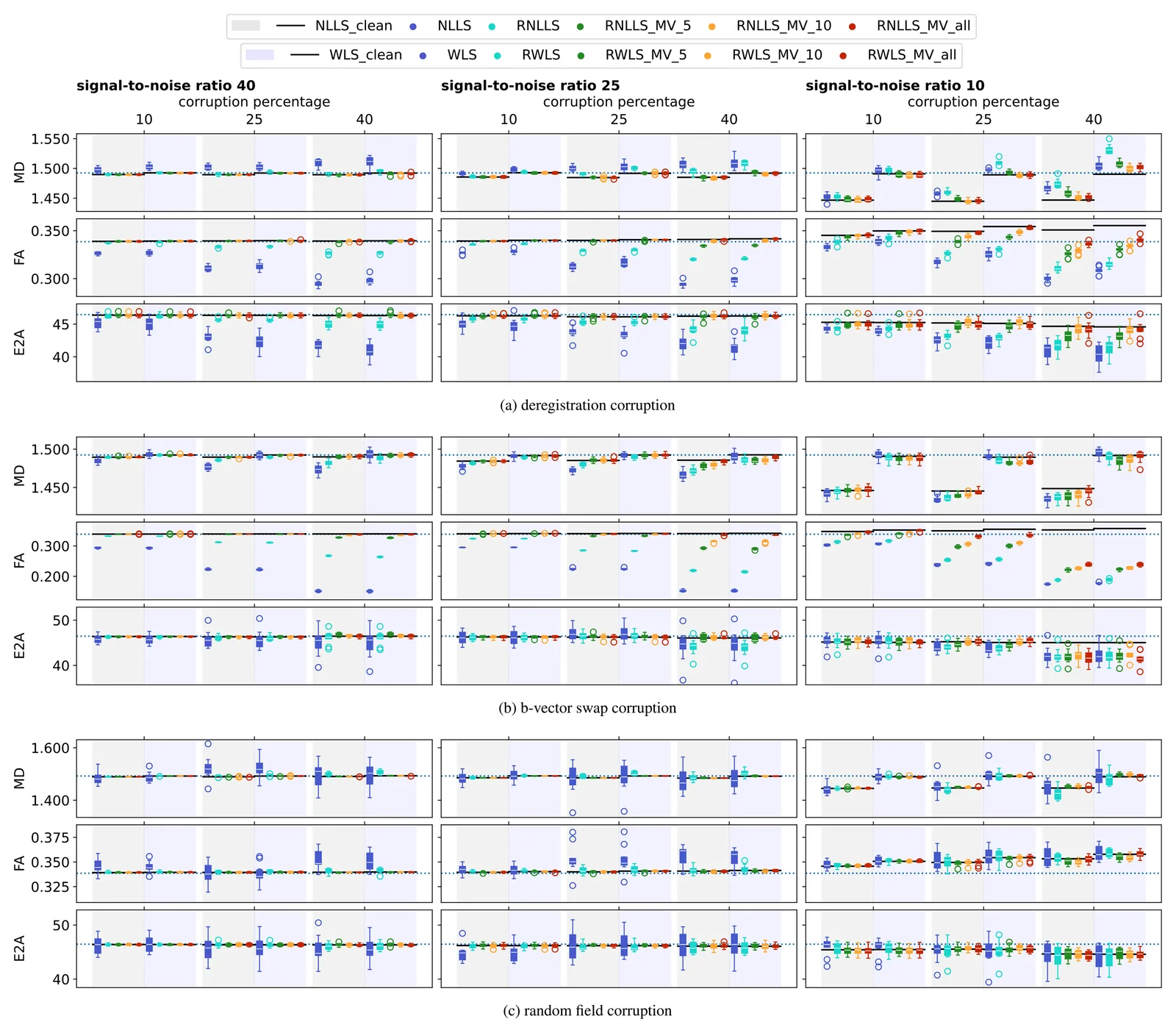
Global diffusion measures MD (×10^−3^ mm^2^ /s), FA, and absolute E2A (degrees) for synthetically corrupted data. Each sub-plot corresponds to a different corruption type. Different percentages of images have been corrupted, and different SNRs have been used to generate Rician error. The dotted line extending across each plot is the “synthetic ground truth” from which synthetic data (with noise and corruptions) was generated, whereas the darker solid lines are the mean of non-robust fits excluding the images that were artificially corrupted. The box-and-whisker plots represent 10 synthetic datasets per corruption configuration (for narrow box-plots, the median line has been removed for clarity). Different colours correspond to different fitting methods, shown in the legend. For each corruption configuration, NLLS and WLS methods are shown to the left and right against a differently shaded background.

**Fig. 7:**
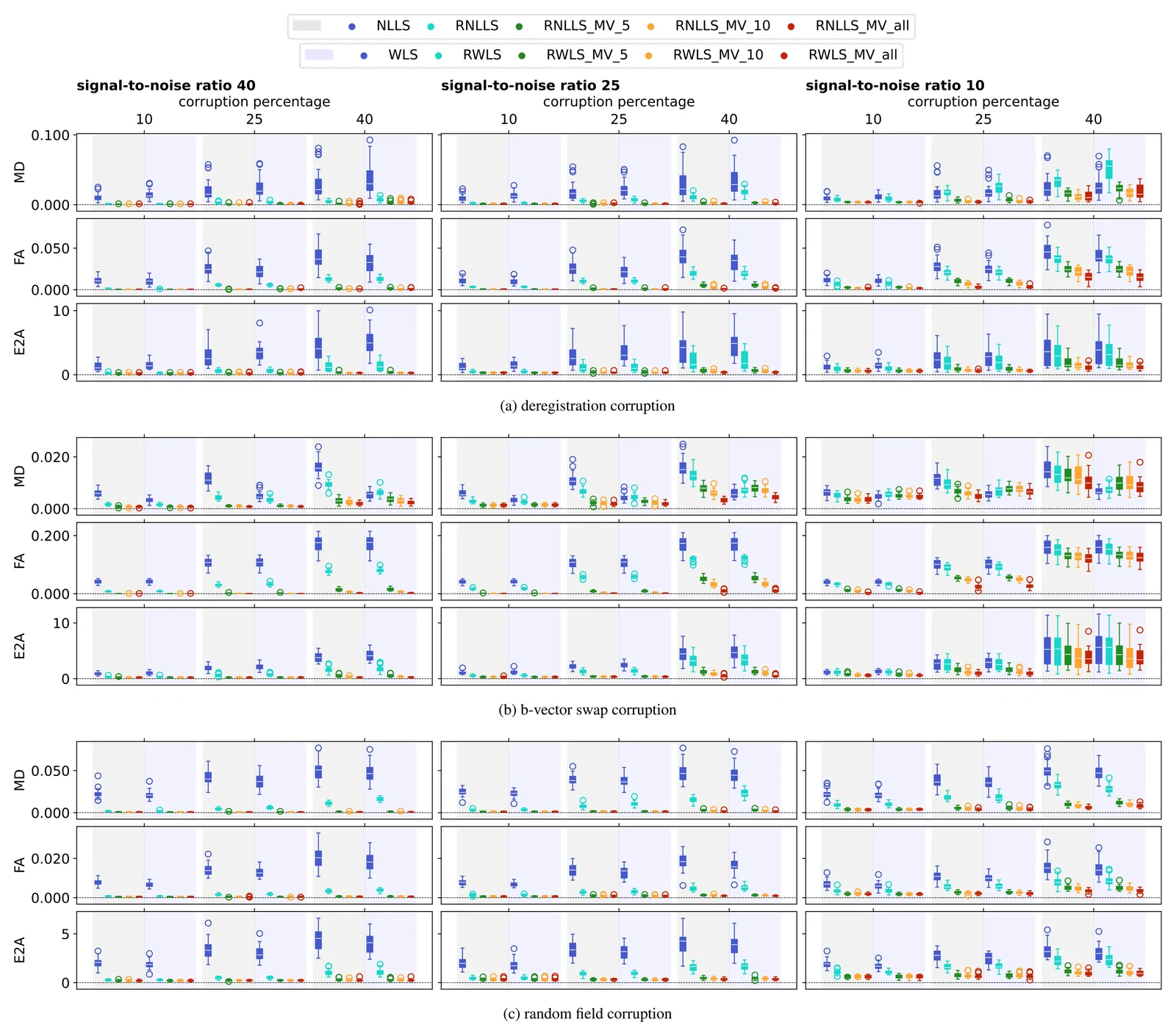
RMSE of global diffusion measures of MD (×10^−3^ mm^2^ /s), FA, and absolute E2A (degrees) for synthetically corrupted data. Each sub-plot corresponds to a different corruption type. Different percentages of images have been corrupted, and different SNRs have been used to generate Rician error. The RMSE is calculated against the mean of non-robust fits excluding the images that were artificially corrupted. The box-and-whisker plots represent the RMSE scores of all subjects (for narrow box-plots, the median line has been removed for clarity). Different colours correspond to different fitting methods, shown in the legend. For each corruption configuration, NLLS and WLS methods are shown to the left and right against a differently shaded background.

Fig. 6 shows, for a single subject, global MD, FA, and absolute E2A, for different fitting methods for deregistration (Fig. 6(a)), b-vector swap (Fig. 6(b)), and random field (Fig. 6(c)) corruptions. This example was a typical volunteer, as determined by comparing metric values against the group values in Fig. 2. The metric results for 10 different synthetic samples are represented as box-and-whisker plots. The median is shown as a white line within each box, except where the box was too narrow so the median line was not drawn for clarity. The results making up each box-and-whisker plot in Fig. 6 can be used to generate a single root mean square error (RMSE) score for each combination of corruption type, percentage, SNR, and fitting method. Fig. 7 is arranged similarly to Fig. 6, but shows RMSE scores of all subjects for each corruption configuration.

Different synthetic corruptions have different effects on the global diffusion measures, and while the effect size varies between subjects, some general results were identified. Unsurprisingly, the diffusion measures deviate more from the synthetic ground truth for increasing levels of corruption in all cases. The ability of all fitting methods to handle corruptions becomes worse for increased levels of corruption and for decreased SNR. The effect of corruptions on non-robust fitting (NLLS and WLS) can be described as follows. Deregistration corruption caused a positive bias in MD, and a negative bias in FA and absolute E2A. b-vector swap corruption produces a small negative bias on MD and a large negative bias on FA. For E2A, there is little bias until large corruption percentages, but there is still large variation in E2A between the synthetic samples. Random field corruption produced relatively little bias on any of the diffusion measures, perhaps because a different field was applied to each corrupted image, rather than fields with a similar pattern. However, the variance of the estimated diffusion measures still leads to an RMSE comparable to the other types of corruptions.

Notably, MVOD performs better than SVOD in all cases. Note that Fig. 7 does not show significance test results, because the significance tests between SVOD and the corresponding MVOD methods were passed in every single case. In general, using MVOD with more voxels results in superior performance, although the difference between a 10 voxel neighbour and using all voxels seems minor. Given that in these examples, we chose subsets of images to corrupt (such that, nearly all myocardial signals in these images will be corrupted), this is not surprising.

SVOD robust fitting performs extremely well for up to 25% corruption for SNR 40, and up to 10% for SNR 25 and 10 (depending on the corruption type). For SNR 10 and 40% corruption, even MVOD methods fail to recover the synthetic ground-truth for b-vector swap corruption given the severity of these corruptions (note that the actual SNR of many signals will be significantly less than the provided nominal value based on average *S*_0_). Importantly, these results are in-fact multi-variate. For WLS methods for b-vector swap corruptions for SNR 10, it seems that robust methods do no-better or slightly worse for MD, however the usual pattern is seen for FA and E2A in which robust fitting is superior, with MVOD beating SVOD.

## 4 Discussion

This work was motivated by the observation that SVOD often failed to detect ‘incorrect’ signals in corrupted images, simply because these signals are not statistically outliers. MVOD is a way to detect more of these incorrect signals. While a method in its own right, it helps to shed light on the question of whether the insensitivity of SVOD is actually important in practice — if MVOD detects most of the missed signals, but makes little difference compared to SVOD, this would suggest to us that the problems with SVOD are relatively unimportant, and cDTI researchers can confidently replace shot-rejection with SVOD. Our results, while showing that robust-estimation with MVOD is more powerful (and, in the present examples on the original data, could in some cases further reduce MD, increase FA, and recover a better helix angle pattern compared to SVOD), seem to confirm that SVOD still works well for the purposes of recovering diffusion parameters, even though many ‘incorrect’ signal may still be present in the fit. Of course, if the original datasets had lower SNR, it is highly likely that the benefits of MVOD over SVOD would become much more important. This could be the case for DKI, for example, in which the SNR at higher b-values is significantly lower, making outlier detection more difficult.

Importantly, SVOD is superior to shot-rejection. To pre-empt the criticism that ‘better’ shot-rejection would nullify this observation, we will just point out that such claims are examples of the ‘no true Scotsman’ fallacy. The fundamental limitation of shot-rejection is that the paradigm of perfectly splitting images into non-corrupt and corrupt sets is flawed: both SVOD and MVOD can detect outliers in images that would be missed by shot-rejection. The fundamental deficiency of SVOD is that it not possible to detect incorrect signals at the single voxel level if these signals are not statistically outliers. Corruptions are usually present at the multiple-voxel level, but not necessarily at the whole-image level. The advantages of robust-estimation as a means to identify outliers is that the entire image series, along with the prior knowledge that the diffusion tensor model (in this case) can model the signal, is used to identify outliers. MVOD simply includes additional prior information: residuals ought to be (mostly) independent, so corruptions can be identified by statistical tests on multiple-voxels in a spatial neighbourhood.

In the context of comparing groups, the differences between fitting methods is still relatively minor. But diagnostically, the differences between fitting methods for an individual subject may be quite important, especially within localized regions of interest. Fig. 3 with non-robust fitting would suggest elevated MD on the septum, however with robust fitting this apparent elevation mostly disappears, even more so with MVOD. Fig. 4 shows that robust fitting methods appear to recover right-handed cardiomyocyte orientation that might have otherwise been inferred to have been missing near the septum. Given our recent work demonstrating that a reduction in right-handed cardiomyocyte population may be a hallmark of HCM (Das et al., 2021), this is a noteworthy observation with potential diagnostic relevance: the right-handed orientation ‘re-appears’ when using robust fitting methods on this subject, proving that its apparent absence was artefactual. Additionally, with the improvement of RMSE with robust fitting as an indicator of poor fit of the model, some regions of elevated MD in Figs. 3 and 4 might be better interpreted as artefacts than as tissue properties.

To our knowledge, this is the first time that an exactly equivalent robust algorithm has been applied to the NLLS and WLS problem for DTI data. What is revealed by this comparison, is that non-robust WLS methods are particularly vulnerable to corruptions, resulting in significantly inflated values of MD in some subjects. The advantageous properties of the WLS estimator for DTI estimation only apply when the data can be modelled as the true signal plus Rician distributed noise, and this is not the case when physiological noise and corruption are involved. Weighting by the signal (even the predicted signal, as done here) when this signal is incorrect due to corruptions and outliers, may lead to substantially worse estimates than NLLS fitting. Therefore, it seems particularly important to use robust fitting with WLS.

## 5 Conclusion

In this work, we have attempted to answer the question of whether robust-estimation can replace shot-rejection in cardiac diffusion tensor imaging, and whether the deficiencies of single voxel outlier detection are important for recovering correct diffusion tensor metrics. We have presented robust fitting with M-estimators followed by single-voxel-outlier-detection and multiple-voxel-outlier-detection. Our results demonstrate that MVOD is more robust than SVOD, particularly for large numbers of corrupted images and low SNR. Nonetheless, the improvement of MVOD over SVOD seems relatively minor for cardiac DTI, even as SVOD gives large improvements over shot-rejection, suggesting that researchers need not worry that SVOD misses signals that would be identified by shot-rejection, even if MVOD could identify these signals. We recommend cDTI to start using robust-estimation in place of shot-rejection.

## Supplementary Material

Appendices

## Figures and Tables

**Table 1:** Table of difference of group means, medians, and p-values, for MD, FA, absolute E2A, for several tested methods. Within each method category (either NLLS or WLS) the largest difference of means and medians are shown in red, and the lowest *p*-value is shown in blue.

Method	MD				FA				E2A		
	mean	med	pval		mean	med	pval		mean	med	pval
NLLS	0.09085	0.064	0.01102		−0.07136	−0.0785	5.762e−05		19.14	22.43	0.00016
NLLS_SR	0.09591	0.0755	0.003319		−0.07003	−0.0745	4.605e−05		19.82	22.44	0.0001311
RNLLS	0.0987	0.07726	0.002041		−0.07331	−0.07933	3.049e−05		20.3	23.95	0.0001311
RNLLS_MV	0.1089	0.08396	0.0008657		−0.07499	−0.08064	3.049e−05		20.28	23.47	0.0001311
WLS	0.08983	0.0535	0.07168		−0.07362	−0.0755	3.761e−05		18.89	22.15	0.0001948
WLS_SR	0.1012	0.052	0.02989		−0.07023	−0.076	3.72e−05		20.01	22.37	0.00016
RWLS	0.0977	0.07915	0.005302		−0.0733	−0.0801	3.782e−05		20.16	24.08	0.0001311
RWLS_MV	0.1086	0.08346	0.001458		−0.07461	−0.0808	3.049e−05		20.23	23.61	0.00016

## Data Availability

We are unable to share the data, but our code is open source.
