# Supplementary material for: Outlier detection in cardiac diffusion tensor imaging: Shot rejection or robust fitting?
Source: Med Image Anal. Author manuscript; Available in PMC 2026 Oct 20. (PMC7619604; doi:10.1016/j.media.2024.103386)
Supplement: Supplementary file 1 [file EMS218948-supplement-Appendices.pdf]

Appendix A. Shot-rejection

Shot-rejection in cardiac DTI can be very time consuming and challenging, because there are usually 3 slices and 100 s of images per slice. While some images can be easily identified as corrupt, one of the main difficulties is determining how much shot-to-shot variation is acceptable. We have developed methods to automate this process using the mean squared error between the predicted image and observed image (Coveney et al., 2023), although this is closer to MVOD than to standard shot-rejection used in cDTI.

For shot-rejection in this paper we calculate the mutual information between the reference image used for registration (the mean of registered b-value 100 s/mm^2 images, themselves registered to a chosen b-value 100 s/mm^2 image) and each image, within the square mask used for registration. For each b-value separately, we used MED(MI) $\pm$ $3 \times 1.4826 \times$ MAD(MI) as a cut-off for image-shot rejection, where MED is median and MAD is median absolute deviation (those beyond the threshold are shot-rejected). This choice makes the shot-rejection reproducible, and works well in datasets where the image quality is high and consistent except for the corrupted images.

Appendix B. Myocardial segmentation

The myocardium was segmented using in-house software in the following ways: (1) epi-cardial and endo-cardial surfaces were defined to segment myocardial tissue, taking care to exclude partial volume effects at the surfaces if necessary; (2) the American Heart Association (AHA) model was used to segment the 3 slices of the myocardium into 16 segments; (3) regions of artefact, such as magnetic field inhomogeneties (which often cause local distortions), fat-shift, etc. are determined such that they can be excluded from final metric statistics (e.g. mean MD, etc.). Such artefacts cannot be compensated for by robust fitting, because the artefacts exist in every image in the series, and corresponding tensor metrics do not represent tissue properties. In our workflow, we utilize both the diffusion tensor metrics, as well as the original image series, and often the CINE short axis series, in order to perform segmentation. Segmentation was performed by an expert and validated and improved in group discussions with collaborators.

Appendix C. The Hat matrix

The variance of fitting residuals is not homoskedastic, and decreases for points with higher leverages. It is common to account for leverages by dividing residuals by the factor $\sqrt{1-h_{ii}}$ where h_{ii} is the i'th diagonal of the Hat matrix for the regression problem. The Hat matrix, also called the projection matrix, links the predicted response to the measured response:

$$\hat{\mathbf{y}} = H\mathbf{y} \tag{C.1}$$

Denoting the design matrix of the linearized problem as X, for the NLLS problem, we utilize the Hat matrix for the (linearized) OLS problem (since to calculate the NLLS leverages explicitly is an intractable problem due to combinatorics):

$$H = X(X^T X)^{-1} X^T \tag{C.2}$$

and for the WLS problem we utilize the Hat matrix involving the weights from the last robustly weighted iteration of the IRLS algorithm:

$$H = X(X^T W X)^{-1} X^T W^T \tag{C.3}$$

where W are the weights for the squared residuals. The experimental designs in DTI are carefully chosen, so while accounting for leverage effects on residuals is generally sensible, it may have relatively little effect in practice.
